# Constructing a diversified online neurology teaching model under the COVID-19

**DOI:** 10.3389/fmed.2022.1071414

**Published:** 2023-01-09

**Authors:** Haojun Yang, Yishu Fan, Zhuohui Chen, Shiyu Zhang, Haiyue Wu, Xinhang Hu, Tong Wu, Mengqi Zhang

**Affiliations:** ^1^Department of Neurology, Xiangya Hospital of Central South University, Changsha, Hunan, China; ^2^National Clinical Research Center for Geriatric Disorders, Xiangya Hospital, Central South University, Changsha, China

**Keywords:** COVID-19, education reform, medicine, neurology, online teaching

## Abstract

**Purpose:**

To construct a diversified and comprehensive network teaching model to provide highly qualified medical teaching in neurology under COVID-19 pandemic.

**Materials and methods:**

Published studies on medical education were systematically reviewed and summarized. Based on previous studies and our experience, we constructed a novel online neurology teaching model and applied it to real scene. Students taking traditional in class lessons and online lessons were asked to finish the test, respectively, to compare the efficiency of learning. Questionnaires were designed and assigned to get the feedback from students.

**Results:**

The average test score of students who take online class (84.27 ± 4.64) was significantly higher than those who take in class lessons (82.08 ± 6.17) (*P* < 0.01). According to the feedbacks from students, online classes were more attractive to students than the conventional one.

**Conclusion:**

Traditional single-mode teaching can no longer meet the needs of current medical education, especially under the rampant epidemic. This novel teaching mode, which orchestrates high-tech tools, diverse teaching methods and traditional teaching concepts, provides the solution to the challenge faced by traditional medical education. We believe that this novel online teaching mode will boost neurology education and inspire educators in other fields during this tough period.

## 1. Introduction

A pneumonia epidemic, first reported in Wuhan, Hubei province in December 2019, has become a global issue because of its surprising infectivity through droplets, air, and physical contact (1). On 11 February 2019, the World Health Organization (WHO) officially named this disease as the coronavirus disease (COVID-19) (2) and the International Committee on Taxonomy of Viruses named the virus as severe acute respiratory syndrome coronavirus 2 (SARS-CoV-2) (3). In this special epidemic period, traditional in class teaching no longer meets the needs for disease control and prevention due to the close social distance. To curb the further spread of virus and consolidate the obtained achievements in epidemic prevention, local governments decided to delay the reopening of schools at all levels. Educational authorities have proposed using online platforms and cloud classrooms for online teaching in the extended holiday to achieve “continuous teaching even when classes are suspended.” With richer teaching resources, more diversified course designs and free of time limits, online teaching has shown huge and magic power in this tough period. Despite all these advantages, there remain some hurdles and difficulties, such as the restrictions on course contents, the rigid demand for digital equipment, the requirement for students’ self-learning ability and most importantly, the loosened ties between students and teachers separated by the cold screen. All these problems will get tougher and trickier when it comes to clinical medical education, as it takes time to record the changes of the patient’s condition and put theory into practice. Therefore, it is of great importance to construct a novel teaching mode in the COVID-19 pandemic to improve the teaching quality for medical students.

Recently, medical education has generally adopted a teaching method of “mainly traditional teaching supplemented with online teaching,” which has gradually replaced traditional “spoon feeding” and has obtained positive feedback. After systematically reviewing the literature, we found that previous models and methods in neurology teaching have their limitations. Considering the dual needs of neurology teaching theory and practice, as well as the difficulties and challenges in comprehensive online teaching during the COVID-19 epidemic, we have constructed a diversified online teaching method for neurology education. We intend to combine online courses, massive open online courses (MOOC) and virtual reality simulation systems to improve the quality of online education. In this article, we took neurology education as an example to summarize the practical experience of online teaching and evaluate diverse online teaching methods during the COVID-19 pandemic. Besides, we combine multiple teaching modes to construct a new diversified online teaching mode to make up for the shortcomings of traditional online teaching. Though developed in response to challenges under COVID-19 pandemic, this teaching mode is also helpful in remote education and tans-national education, which can be further applied in the future.

## 2. Study participants and methods

### 2.1. Design

We systematically reviewed the published papers involving in online education and summarized the characteristics of online teaching, neurology education and existing neurology teaching methods. Based on this, we established a new teaching model and applied it to the neurology teaching of undergraduates in Xiangya Medical College of Central South University. Students taking online and traditional offline classes were asked to finish tests to evaluate the neurology learning effectiveness. Questionnaire surveys were conducted to obtain their feedback on the new teaching model.

### 2.2. Participants

We chose 260 clinical medical undergraduates of Xiangya School of Medicine, 130 of them taking online courses and others taking offline courses. Forty hours were allocated to theoretical and practical teaching, twenty hours for each. After 6-month classes, from February to August 2020, they were asked to finish the test. The study was approved by the ethics committees of the Xiangya Hospital, Central South University. The purpose of the study was clearly explained to the participants before distributing the questionnaire. Informed consents were obtained, and all questionnaires were administered anonymously.

### 2.3. Teaching methodology

**FIGURE 1:**
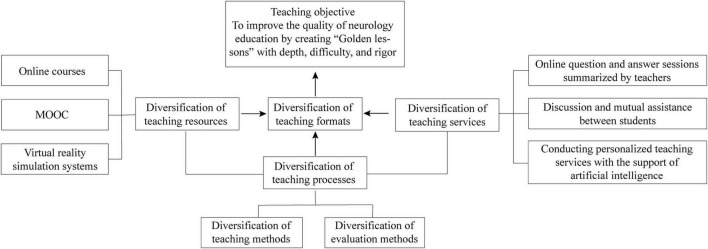
Construction of a diverse online neurology teaching method during the COVID-19 pandemic.

We constructed a diversified online teaching method in neurology education based on three aspects: resources, processes, and services, aiming to overcome the current challenges and improve neurology education quality during the COVID-19 pandemic and for future education (Figure 1). This teaching mode emphasizes on the subjective motivation and initiative of the students and interdisciplinary cooperation of teachers.

#### 2.3.1. Construction of diverse teaching resources

##### 2.3.1.1. Online courses

Images, videos, and animations were used in the theoretical online teaching. Continuous image photography, audio, and short videos of positive signs of classical neurological disorders were obtained from the Department of Neurology, Xiangya Hospital, Central South University and online education resources. Those sources were used to construct and enrich the positive nervous system sign image and video library (4), which greatly arouses the interest of students and helps them to deepen their theoretical knowledge without leaving home.

##### 2.3.1.2. MOOCs

Since its launch in 2008, MOOCs have become a prominent representation of remote online teaching. The emergence of MOOCs has enabled the conversion of teaching mode from “first teach then learn” to “first learn then teach” (5). Taking neuroanatomy knowledge as an example, students had learned systemic and local anatomy during the basic medicine phase but have almost forgotten about them by the time they were enrolled for the neuroanatomy courses. However, with the help of MOOC, they can review those knowledges online, bridging the knowledge gap and receiving a more efficient learning.

##### 2.3.1.3. Virtual reality simulation systems

Medical history collection and physical examination are indispensable for localization and qualitative diagnoses in neurology. After teachers have displayed online videos of neurological examinations for students, students can then enter the virtual reality system and select different types of neurological diseases for online simulation (6, 7). Then students are required to make a corresponding diagnosis based on the medical history and physical examinations.

#### 2.3.2. Construction of diverse teaching services

##### 2.3.2.1. Online question, answer, and summary from teachers

Professors are responsible for giving neurology theory while teaching assistants are responsible for the summary and the question-and-answer sessions after online courses. The teaching assistant should clearly know students’ questions and provide targeted and personalized guidance for them to solve the problems. Carefully avoid directly giving them the answers. They must help students understand the problems in the diagnostic process and assist them in developing clinical thinking. In addition, heuristic guidance is needed to open their minds and give them timely feedback. The teaching assistants can also use video conference to periodically summarize knowledge in various diseases.

##### 2.3.2.2. Discussion and mutual assistance among students

Discussions around the central topic give students the opportunities to voice their own opinion and open their minds. By doing so, they can learn from each other and have a better teamwork.

##### 2.3.2.3. Personalized teaching services with artificial intelligence

Students’ learning foundation, capability, and interest are quite different. With the help of artificial intelligence, teachers can get a clearer understanding of students’ learning situation through questionnaires and tests, and further provide personalized guidance.

#### 2.3.3. Construction of diverse teaching processes

##### 2.3.3.1. Diversification of teaching methods

Before the course, teachers should clearly state the learning objectives. Besides cold knowledge, warm empathy and humanistic care are also essential for medical students (8). Teachers can employ CBL, PBL, or flipped classrooms to develop the initiative of students so that they can improve autonomous learning competency within a limited timeframe. In our new teaching mode, to make the clinical neurology teaching diversified and personalized, teachers make and apply microvideo chips (10–15 min) for scenario or case simulations, which hides professional knowledge (9). Students are asked to discuss these cases based on groups, which helps them to develop clinical thinking and grasp professional knowledge better.

##### 2.3.3.2. Diverse assessment methods

The Plan-Do-Check-Act (PDCA) cycle is currently regarded as one of the most high-quality management methods and is widely used in medicine. PDCA can improve medical processes and standardize hospital workflow (10–12). The entire PDCA cycle reflects the objective law of “understand-practice-re-understand-re-practice” in human objective cognition. It can also serve as a problem-solving mindset. Actually, teaching evaluation is a type of feedback on students’ acceptance of the teaching method and content, which is equivalent to the re-understanding phase in the PDCA cycle. Teaching evaluation cannot be divorced from a rational evaluation system and a suitable evaluation entity. Besides, student evaluation from teachers, student’s self-evaluation and mutual evaluation can never be ignored as well. In addition, evaluations of teachers and their teaching methods from students are also indispensable as they can point out the problems in the ongoing teaching mode. In our teaching process, we regularly obtain feedback from students through questionnaires, and accordingly make improvements and adjustments to the teaching model.

#### 2.3.4. Specific implementation processes

##### 2.3.4.1. Active learn before class

We established an online chat group for the communication between students and teachers during the teaching process. The teaching resources of neurology were delivered to the students before class through the online communication. Students watched the videos and other learning resources based on the learning objectives and requirements released by teachers in advance. Before the lesson, students’ interest and their prior knowledge will be understood by means of pre-class test, quizzes, anonymous voting, group discussions, etc., so as to adjust the depth and progress of the following teaching content. We use a test including about 10 choice questions to evaluate the mastery of teaching content before class.

##### 2.3.4.2. Discuss in class

In class, the professor first gives a lecture to introduce typical cases in the form of text, PPT, pictures, videos, etc., to attract students’ attention and arouse their curiosity and interest in learning. Typical cases can be presented in a variety of ways, including but not limited to videos and patients simulated by doctors. Then they should set clear, appropriate, achievable and measurable learning objectives from the perspective of students to help them understand the point of this class. The problems discussed in class should be diverse and vivid, so as to improve students’ interest in learning. in addition, teacher-student and student-student interactions are encouraged to help the students learn the knowledge better. These processes are conducted through Wemeet (an online meeting application).

##### 2.3.4.2. Review after class

**FIGURE 2:**
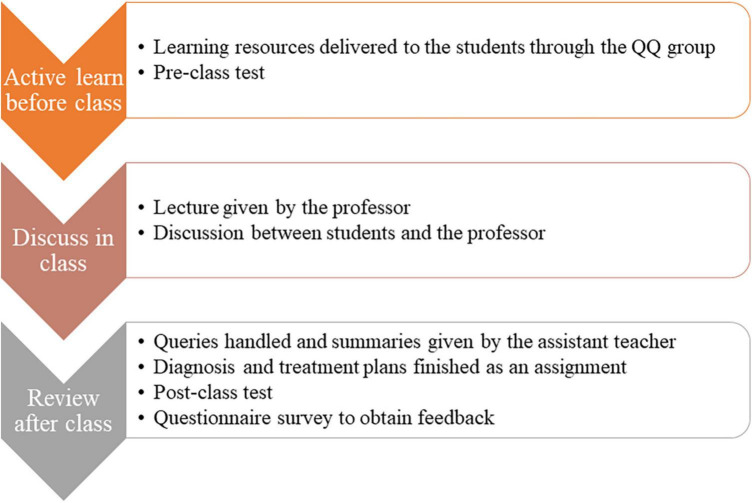
The diagram of the teaching process under the diverse online neurology teaching mode.

After class, students are asked to participate in the learning activities in a variety of interesting ways, such as personal reports, group discussions, case studies, role plays, and storytelling, so as to deepen the students’ understanding and impression of what they have learned and improve their interest in learning. Students can ask questions and the assist teacher answers their questions and gives a summary through the online chat group. A task will be delivered to students, and they are asked to independently finish a diagnosis and treatment plan, including auxiliary examination, diagnosis, differential diagnosis, treatment, prognosis evaluation and preventive measures, by consulting literature and reviewing textbooks. Besides, a test including about 10 choice questions is used to evaluate the learning effectiveness of neurology after class. The test was designed by the professors of department of neurology. A questionnaire survey was conducted after that semester to obtain the attitudes from students toward the diversified online teaching method for neurology. The specific implementation processes can be seen in Figure 2.

### 2.4. Research methods

We used Wenjuanxing (an online website for questionnaire survey) to investigate the attitude of the students for this new online teaching method. The responses of the survey were scored using a 5-point Likert scale ranging from strongly disagree to strongly agree. Besides, we compared the average test scores of students who take online classes and those who take in class lessons.

### 2.5. Statistical analysis

The data were coded, entered, and analyzed using the SPSS statistical package, version 25.0 (SPSS Inc., Chicago, IL). The test scores were presented as means ± standard deviations (SD). An independent sample *t*-test was used to compare mean test scores between pre-class and post-class. *P* < 0.05 is considered statistically significant.

## 3. Results

**FIGURE 3:**
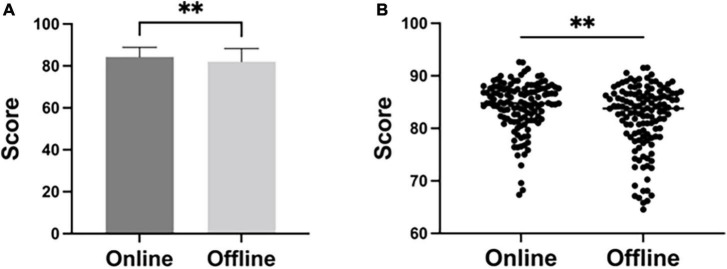
The average test scores of students taking online classes (*n* = 130) and taking offline classes (*n* = 130), analyzed by *T*-test. **P < 0.01.

As shown in Table 1 and Figure 3, the average test score of students who take online classes (84.27 ± 4.64) were significantly higher than those who take offline classes (82.08 ± 6.17) (*P* < 0.01).

**TABLE 1:** The average score of the test.

Groups	The number of students (M/F)	Test score (mean ± SD)
Online	130 (68/62)	84.27 ± 4.64**
Offline	130 (65/65)	82.08 ± 6.17

**P < 0.01 vs. offline group.

M, male; F, female; SD, standard deviation.

**FIGURE 4:**
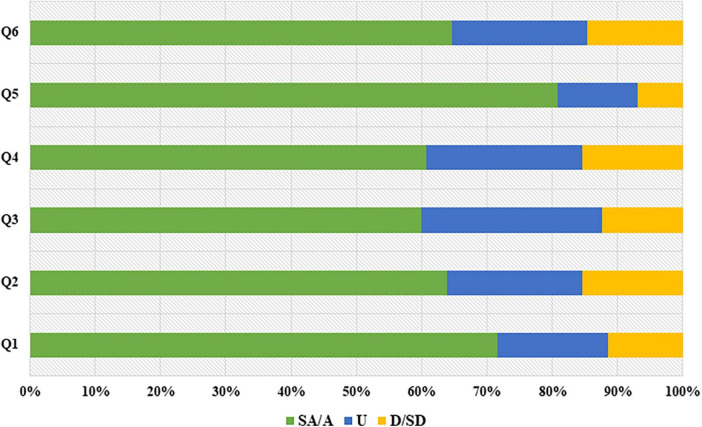
Students’ attitudes toward this novel online teaching mode (*n* = 130). SA, strongly agree; A, agree; U, uncertain; D, disagree; SD, strongly disagree. Q1 to Q6 represent the 6 questions in Table 2.

One hundred and thirty copies of questionnaires were sent out and one hundred and thirty were received with a coverage rate of 100%. The questionnaire results were shown in Table 2 and Figure 4. There were 71.54% (93/130) students liked this online teaching mode and 63.85% (83/130) students believed that this teaching model was innovative. 60% (78/130) of the students thought the online teaching aroused their interest in the study of neurology. In all, 64.62% (84/130) students thought it a good way of teaching.

**TABLE 2:** Students’ attitudes toward this novel online teaching mode.

Questions	SA	A	U	D	SD
I like this online teaching.	53 (40.77%)	40 (30.77%)	22 (16.92%)	7 (5.38%)	8 (6.15%)
The online teaching model is innovative.	29 (22.31%)	54 (41.54%)	27 (20.77%)	9 (6.92%)	11 (8.46%)
The online teaching arouses my interest in neurology.	38 (29.23%)	40 (30.77%)	36 (27.69%)	11 (8.46%)	5 (3.85%)
The online teaching provides a more three-dimensional experience for my learning.	35 (26.92%)	44 (33.85%)	31 (23.85%)	10 (7.69%)	10 (7.69%)
I like the interactive way of online teaching.	59 (45.38%)	46 (35.38%)	16 (12.31%)	5 (3.85%)	4 (3.08%)
The online teaching is a good way for learning and teaching.	40 (30.77%)	44 (33.85%)	27 (20.77%)	7 (5.29%)	12 (9.23%)

SA, strongly agree; A, agree; U, uncertain; D, disagree; SD, strongly disagree.

## 4. Discussion

Online learning is a novel and rapidly growing field for undergraduates, graduate students and healthcare providers (13). In this article, we summarized online teaching methods for neurology and pointed out shortcomings in existing methods based on the teaching characteristics of neurology. We also pointed out and analyzed challenges in total online neurology teaching during the COVID-19 pandemic and constructed a student-centric, diversified and total online teaching method in neurology education to allow teachers to reform their current teaching practices while adhering to traditional teaching concepts.

We evaluated the effect of our mode through post-class tests and questionnaire survey. The results of tests indicated that the scores of students who take online classes were significantly higher than those who take offline classes (*P* < 0.01). Probably reason could be that online classes offer more opportunities for students to acquire and consolidate their lessons via reviewing and relearning. In addition, online teaching via pictures and videos could deepen students’ understanding of specialized diseases. This helps to cultivate their clinical abilities and advanced thoughts on solving complex diseases based on the learned theoretical knowledge and skills. Moreover, diversified teaching forms could promote students’ interest in neurology, their communicational skills and humanistic feelings at the same time. By learning neurology through this diversified online teaching mode, students deeply mastered the teaching content. According to the feedbacks from students, online classes were more attractive to students than the conventional one. It is worth noting that, online learning put forward higher requirements for the autonomous learning abilities of students.

As the population ages, the need for neurologists increases. However, the number of medical students going into neurology cannot meet this increasing need. This is somewhat associated with neurophobia (9), i.e., fear of neuroscience and clinical neurology, and the inability to apply basic neurological knowledge to clinical practice (14). Lin et al. recently published a paper in Neurology, which emphasized the challenges in clinician education and encouraged educators to teach “the art of neurology” (4). How to improve neurology education to make it a “golden lesson” with depth, difficulty and rigor? It is a question that requires serious consideration (15). Neurology has a higher demand for students in clinical thinking and practical skills, which are mainly presented in the following two aspects: (1) The highly required combination of clinical and basic sciences: The complicated neuroanatomical structures, diverse symptoms, obscure etiologies, and similar clinical manifestations in different diseases increases the difficulty in learning neurology. Comprehensive medical history collection and physical examination are powerful tools to determine the site (localization diagnosis) and nature (qualitative diagnosis) of the lesion for diagnosis and differential diagnosis, which requires a clear and logic clinical thinking (10). The neurology education puts forward a dual test in the master of both clinical and basic knowledge for students, and the models of both theoretical (including neuroanatomy, neurophysiology and neuroimmune) and clinical (including clinical manifestations, signs and diagnostic principles) teaching for teachers. (2) Cross-disciplinary training: With the rapid development of medical imaging, medical examinations, neuroimmunology, and neurogenetics, clinical neurology is dynamically growing. This requires educators to cultivate more talents in this field with logical clinical thinking and cross-disciplinary ability to innovate and develop this fast-changing field (16).

Educators and scientists around the world are constantly exploring a better online teaching mode for neurology education. Safdieh et al. (17) provided useful resources for medical students, including a streamlined list of symptom complexes, an abundant list of recommended clinical encounters combined with midrotation feedback. Chen and Evans (9) constructed a MOOC-based flipped classroom model for neurology, bearing “students as a learning entity” in mind. They employed images, flash animations, and videos in online classes and had face-to-face interactions between teachers and students in offline classes. The combination of online and offline education not only ignites the students’ interest in neurology but also promotes their self-learning ability and teamwork spirit, which compensates for shortcomings in the single problem-based learning (PBL) and case-based learning (CBL). Zhou et al. (18) incorporated online assignments, question and answer discussions, and knowledge summaries into offline courses to provide targeted teaching based on the individualized learning situation. Ding et al. (19) proposed the concept of micro-lectures to establish a teaching mode. In their project, traditional neurology teaching is combined with a micro-lecture so that students can get access to the knowledge unavailable in the traditional classroom through online courses. As a complement to traditional teaching, micro-lectures are widely used in basic medical education (20–22). However, its application in clinical education and practice looks dim nowadays as it fails to cultivate clinical thinking and skills in medical students.

With the advent of mobile internet, cloud computing, big data, and artificial intelligence, online teaching has become an important part of the modern education (23, 24). The fusion of information technology, such as MOOCs, flipped classrooms and high-quality network resources has gradually replaced traditional “spoon feeding” teaching mode and become today’s new trend (9). Compared with conventional classroom teaching, online teaching has the following characteristics: (1) Flexibility: online teaching is free of time and space limitations as both teachers and students can flexibly schedule their time to give or have a lecture (25); (2) Personalization: students can pause, advance, and replay the course video according to their own learning situation, which greatly elevates their study efficiency (26); (3) Abundant teaching resources: teaching images and video libraries are timely updated and available to all users (15); (4) Reduced costs: teachers can learn from each other to improve their teaching skills (27). Besides, online lessons could receive support from ads and charities, which reduces the teaching cost and ensures the teaching quality at the same time.

Conducting rational and effective online teaching remains a challenge. It is true that online teaching can be used for basic medical classes. Students who accomplish the tasks in different chapters will have a good grasp of theoretical knowledge. However, the clinical practice is of the same importance in their medical study, such as clinical operations, clinical thinking and physician-patient communication (28). Taking neurology study as an example, medical history collection and physical examination are indispensable for localization and qualitative diagnoses. Besides, lumbar puncture, the most common neurological procedure, requires enough practice to be acquired. In addition, online teaching requires more financial and technique support while many universities lack the required infrastructure. Further, online teaching has a higher requirement not only for the students’ self-discipline and self-learning ability, but also for teachers’ teaching method and quality.

This diversified online teaching mode makes up for the shortcomings of traditional online and offline teaching, explore the subjective initiative in students and fill their knowledge gaps. Besides, it increases students’ interest in learning, enables them to make personalized studying plans, provides them with abundant online exercises and attract them to choose online study. Diversified teaching and evaluation methods further deepen teachers’ understanding of students and contribute to the improvement in previous teaching methods. Therefore, we believe that neurology teaching under this diversified teaching forms can improve students’ autonomous learning and collaborative abilities, which helps them to truly internalize theoretical knowledge, to analyze and solve clinical cases better and to integrate into the clinical atmosphere. The diversified online teaching mode is also of reference and promoting value in ensuring students’ normal courses and continuing education in the face of sudden public health and safety emergencies.

## 5. Conclusion

In general, we constructed a diverse online neurology teaching method by combining advantages of modern information technology, scientific research, and online teaching. Our newly constructed teaching model is proven to be effective in neurology education under COVID-19 pandemic and has received recognition from students.

Moving forward from this pandemic, to maximize the benefits of offline and online teaching and to improve the efficiency of medical education in the future, we recommend that medical schools adopt a combination of both in calss and online teaching. Online teaching should be regarded as a supplement to, not a substitute for, traditional in class teaching. Further research is needed to explore the effectiveness of this novel and diversified teaching mode in remote education and tans-national education. We expect that this online teaching method can be further integrated into traditional medical education, not just in neurology, but also in other disciplines. This will combine the technological strengths with teaching strengths and achieve collaboration among networks, scientific research, and medical teaching to cultivate outstanding medical talents with outstanding clinical skills.

## Data availability statement

The original contributions presented in this study are included in the article/supplementary material, further inquiries can be directed to the corresponding author.

## Ethics statement

The studies involving human participants were reviewed and approved by the Ethics Committee of Xiangya Hospital, Central South University. The patients/participants provided their written informed consent to participate in this study.

## Author contributions

MZ conceptualized the study, acquired the funding, and administered the project. HY and YF wrote the original draft. YF and ZC provided the resources. HW, XH, and TW worked on validation. All authors reviewed and edited the manuscript.
